# Comparative Analysis of LLMs in Dry Eye Syndrome Healthcare Information

**DOI:** 10.3390/diagnostics15151913

**Published:** 2025-07-30

**Authors:** Gloria Wu, Hrishi Paliath-Pathiyal, Obaid Khan, Margaret C. Wang

**Affiliations:** 1 Department of Ophthalmology, School of Medicine, University of California, San Francisco, CA 94143, USA; 2 Department of Biological Sciences, Halmos College of Arts and Sciences, Nova Southeastern University, Fort Lauderdale, FL 33328, USA; hp616@mynsu.nova.edu; 3 College of Osteopathic Medicine, California Health Sciences University, Clovis, CA 93612, USA; khan2504@chsu.edu; 4 Department of Psychology, Harvard University, Cambridge, MA 02138, USA; margaretcwang@post.harvard.edu

**Keywords:** large language models, dry eye syndrome, healthcare disparities, cultural sensitivity, health equity, Grok, ChatGPT, Gemini, Claude.ai, Meta AI

## Abstract

**Background/Objective:** Dry eye syndrome affects 16 million Americans with USD 52 billion in annual healthcare costs. With large language models (LLMs) increasingly used for healthcare information, understanding their performance in delivering equitable dry eye guidance across diverse populations is critical. This study aims to evaluate and compare five major LLMs (Grok, ChatGPT, Gemini, Claude.ai, and Meta AI) regarding dry eye syndrome information delivery across different demographic groups. **Methods:** LLMs were queried using standardized prompts simulating a 62-year-old patient with dry eye symptoms across four demographic categories (White, Black, East Asian, and Hispanic males and females). Responses were analyzed for word count, readability, cultural sensitivity scores (0–3 scale), keyword coverage, and response times. **Results:** Significant variations existed across LLMs. Word counts ranged from 32 to 346 words, with Gemini being the most comprehensive (653.8 ± 96.2 words) and Claude.ai being the most concise (207.6 ± 10.8 words). Cultural sensitivity scores revealed Grok demonstrated highest awareness for minority populations (scoring 3 for Black and Hispanic demographics), while Meta AI showed minimal cultural tailoring (0.5 ± 0.5). All models recommended specialist consultation, but medical term coverage varied significantly. Response times ranged from 7.41 s (Meta AI) to 25.32 s (Gemini). **Conclusions:** While all LLMs provided appropriate referral recommendations, substantial disparities exist in cultural sensitivity, content depth, and information delivery across demographic groups. No LLM consistently addressed the full spectrum of dry eye causes across all demographics. These findings underscore the importance for physician oversight and standardization in AI-generated healthcare information to ensure equitable access and prevent care delays.

## 1. Introduction

Dry eye syndrome affects 16 million Americans and costs the healthcare system USD 52 billion annually, while another 50.2 million Americans remain undiagnosed and have not received care from appropriate eye specialists [1,2]. The condition becomes more common as people age—it affects 8.4% of people under 60, 15% of those aged 70 to 79, and 20% of people over 80 [3]. Patients experience red and painful eyes, foreign body sensation, and blurred vision that interfere with daily activities [4,5]. The condition’s complexity extends beyond surface symptoms, as it frequently coexists with systemic diseases like Sjögren’s syndrome, rheumatoid arthritis, and thyroid disorders—underlying conditions that are rarely tested for during routine optometry visits [6]. Contributing factors include numerous medications such as antihistamines, antidepressants, chemotherapy, and immunotherapies, as well as environmental elements including low humidity, air conditioning, seasonal allergies, and prolonged exposure to computer screens [7].

Despite the prevalence of dry eye, eye care specialists are often inconsistent with their treatments. While there are practice guidelines, the diagnoses of the underlying cause may be inaccurate or incomplete. Primary care physicians, corneal specialists, general ophthalmologists, and optometrists all treat the same symptoms with different outcomes [2].

Many eye care professionals view dry eye disease as a collection of mild symptoms, and treatment is aimed at symptom relief versus etiology [6]. The standard treatment follows a step-by-step process that begins with artificial tears, warm compresses, and anti-inflammatory medications such as cyclosporine A and lifitegrast. Later, additional procedures may be considered, such as lacrimal punctal plugs, heat-based thermal treatment, or biotissue contact lenses when other treatments fail [8]. However, patients may not respond well to these traditional approaches, particularly when meibomian gland dysfunction (MGD) is the underlying cause. Since MGD occurs when these glands become inflamed and blocked, alternative treatments like in-office thermal therapy can help patients by directly targeting gland function and improving symptoms [9]. These heat-based therapies work by liquefying thickened meibum and helping it flow more easily through the application of controlled thermal energy to the eyelids. By specifically targeting MGD, these systems relieve ductal blockages and restore normal gland function. Available thermal pulsation systems include LipiFlow (Johnson & Johnson, Morrisville, NC, USA), Systane iLux (Alcon, Dallas, TX, USA), TearCare (Sight Sciences, Menlo Park, CA, USA), and MiBoFlo (MiBo Medical, Plano, TX, USA) [10].

Many patients seek symptom relief at the drugstore and visit primary care specialists or optometrists before consulting ophthalmologists and corneal specialists [6]. Additionally, there are no standard educational materials, leading to patient confusion and poor treatment adherence [6].

This information gap drives patients to seek answers online through search engines and digital health resources. Google processes over 13.7 billion queries daily, with 7% related to healthcare, amounting to 77,000 healthcare queries per minute [11]. Google has a large volume of 378 million queries every hour or 6.3 million per minute [12].

Large Language Models (LLMs) address this need by responding instantly to medical queries regardless of geographic location, socioeconomic status, or healthcare barriers. All these LLMs have training datasets; some use open-source codes, and some are proprietary.

Different LLMs are trained in different ways, which affects how well they handle healthcare information. Open-source models like Meta’s Llama (Large Language Model Meta AI) and Mistral AI utilize public datasets, including PubMed Central, Wikipedia’s medical pages, and free medical textbooks [13]. These open-source models enable researchers to check and improve the quality of medical training information, and the nature of these models ensures that no single company controls healthcare information [14]. Private models like OpenAI’s GPT-4, Google’s Gemini, and Anthropic’s Claude.ai use carefully selected datasets that incorporate licensed medical databases and peer-reviewed journals, which can provide more accurate and detailed information. However, exact data sources are not disclosed [15].

Grok (xAI, San Francisco, CA, USA), a new LLM, uses Reinforcement Learning from Human Feedback (RLHF) and explicitly incorporates data from X (formerly Twitter) feeds, some of which contain profanity and slang [16]. X has 600–700 million monthly active users, driving engagement to Grok, which has a total of 35 million monthly active users [17,18].

Content from X users may come from bots or accounts funded by corporations, foreign governments, or other groups seeking to disseminate false health information. Research from the University of Indiana found that 6% of bot accounts spread 31% of unreliable information on the platform [19]. Training AI (Artificial Intelligence) models on unfiltered social media content raises concerns about how everyday language with slang, profanity, and cultural references might affect health information delivery. Grok uses techniques such as Proximal Policy Optimization (PPO) and RLHF during development, which help align the model with human preferences [20].

The release of ChatGPT (OpenAI, San Francisco, CA, USA) in November 2022, with natural language responses, led to 100 million users one month after its launch. Currently, ChatGPT has 601.5 million unique visitors generating approximately 5.19 billion visits monthly. The user base is 64.32% male and 35.68% female, with more than 45% of users under age 25 [21]. Google processes over 13.7 billion queries daily, 7% of which are health-related or 77,000 healthcare queries per minute [11].

Google’s Gemini (Alphabet, Mountain View, CA, USA) serves approximately 350 million unique monthly users and focuses on factual accuracy by connecting to Google’s massive database that handles over billions of queries daily. The platform trains on images, voice recordings, and digital data and rapidly grew from zero to 30 million monthly active users, gaining its first million users in just five days [21,22]. Unlike traditional AI models, Gemini uses a special neural node system based on DeepMind technology that processes information through interconnected nodes that adjust based on the information they handle. This flexible system can access multiple knowledge areas simultaneously, similar to how the human brain works, using DeepMind and Pathways Language Model 2 (PaLM 2) architecture for advanced multimodal AI processing [20].

Claude.ai (Anthropic, San Francisco, CA, USA) was created in 2023 and has 18.9 million monthly users. It uses a special training method called “Constitutional AI” that teaches it to be “helpful”, “safe”, and “honest”—which is important for health topics [23,24]. Unlike other AI systems that only learn from human feedback, Claude.ai follows clear ethical rules that guide how it answers sensitive health questions. The system writes responses, checks them against these rules, and fixes them multiple times to reduce harmful or wrong health information [24,25]. Claude.ai responds to millions of health questions by providing educational information while clarifying that its responses do not constitute medical advice.

Meta AI (Meta, Menlo Park, CA, USA) launched on 27 September 2023, and quickly grew to over 500 million monthly users by using Meta’s extensive collection of user interactions, public posts, and shared content while protecting user privacy through anonymization [26]. The platform operates on Meta’s own Llama technology, specifically the massive Llama 3.1 model with 405 billion parameters, making it one of the largest open-source language models available in three sizes: 8 B, 70 B, and 405 B parameters [27]. Meta AI can handle text, images, and voice inputs across all of Meta’s apps and uses RLHF with a special focus on supporting multiple languages for Meta’s global user base of over 3 billion people [28]. Meta AI stands out by working smoothly across all Meta apps while maintaining user privacy and offering personalized assistance.

## 2. Methods

This study used the most commonly used LLMs: Grok, ChatGPT, Gemini, Claude.ai, and Meta AI. The most advanced models of each LLM utilized were: Grok 3, ChatGPT 4.0, Gemini 2.5 Pro, Claude Sonnet 4, and Meta AI (Llama 4).

Grok was included as the newest LLM with unique training from social media conversations. ChatGPT was included as the most widely recognized AI chatbot, serving as the standard for consumer expectations. Gemini was selected due to Google’s dominance in health-related searches, which processes billions of health questions daily. Claude.ai was selected for its safety-focused design approach, emphasizing ethical healthcare information delivery. Meta AI represents the most extensive user base, integrating Facebook, Instagram, and WhatsApp platforms.

Each LLM was analyzed for its capabilities and differences in providing health information. To ensure a comparative analysis, all five models were evaluated using standardized prompts across eight demographic categories representing different ethnic and gender groups. Groups included in the query included hypothetical male and female patients of White, Black, East Asian, and Hispanic races. For the Hispanic queries, the terms “Latino” and “Latina” were used as more casual replacements, which would better simulate conversation. There could also be a difference between using these terms and the more professional “Hispanic male/female”.

The following template was used to query the LLMs:

1. “I am a 62-year-old [race] [male/female] and I have painful red eyes that feel dry all the time. I take no medications, and I do not wear contact lenses. What should I do? Who should I see?”.

The patient, being 62 years old, provides contextual assessment for LLMs when creating a response. Research identifies five factors influencing prompt effectiveness: user intent, model understanding, domain specificity, clarity and specificity, and constraints [29]. A complementary framework proposes five design principles: concise, logical, explicit, adaptive, and reflective prompts [30]. These frameworks demonstrate that prompt construction affects response quality, supporting our single-query methodology to prevent artificial refinement and biases. Repeated queries within the same session can lead to “training the chatbots” to give the “correct” response, called priming [31]. After multiple prompts, the metrics that control the LLMs will lead it to create an artificially enhanced response [32,33]. In real-world clinical scenarios, patients do not repeatedly ask the same query multiple times, making our single-query methodology more representative of actual patient-LLM interactions and ensuring ecological validity of our findings.

We chose age 62 because dry eye problems become more common as people age. Research indicates that over 75% of individuals aged 65 and older experience some dry eye symptoms [4].

At 62, both males and females are at peak risk for developing dry eye disease. This is particularly pronounced in women following menopause (average onset age 52), as hormonal changes impair tear production [34]. Additionally this demographic has higher rates of comorbidities necessitating medications that can induce dry eye symptoms [4].

Another reason we chose the age of 62 years is that they are increasingly using online health resources and AI chatbots to look up their symptoms before consulting a doctor. This makes them perfect for studying how AI provides healthcare information [35].

We measured the word count in each AI chatbot’s responses to evaluate how much information different racial groups received. We aimed to investigate whether certain racial groups consistently received longer, more comprehensive answers while others received shorter, less detailed responses. Significant disparities in response length could indicate potential bias in how AI systems provide healthcare information across different demographic groups.

We used the Flesch-Kincaid Grade Level (FK) which indicates the U.S. school grade level required to understand the text [36,37]. These tests gave us a fair way to measure how easy the language was to read and how complex the sentences were. This lets us directly compare how well the models created readable text. The U.S. Department of Education uses the FK test to assess the readability of educational materials. The formula tells us the “average number of years of education” usually needed to understand the text. The formula is [37]:

Grade Level = 0.39 × (words/sentences) + 11.8 × (syllables/words) − 15.59

Research on dry eye educational materials reveals that online patient resources often exceed the recommended sixth-grade reading level, creating comprehension barriers that can lead to inadequate disease understanding and adverse health outcomes among patients seeking information about dry [38].

We measured response times for each LLM using a manual stopwatch. Time delays may impact user engagement for healthcare questions among different ethnic groups. Longer response times cause users to lose patience and leave before receiving complete medical guidance or become distracted and only skim the advice instead of reading it carefully. Response time variations between ethnic groups may indicate system biases. This is especially concerning in urgent health situations where users need rapid information to make critical medical decisions.

We also measured how similar the answers were using cosine similarity score (CSS). A score of one (100%) meant the answers were the same [39].

For evaluating cultural differences, we used the paid version of ChatGPT 4.5. We scanned the response from the five LLMs, (Grok, ChatGPT, Gemini, Claude.ai, Meta AI) and asked ChatGPT-4.5 to evaluate how well each was tailored to different demographic groups.

ChatGPT-4.5 scored cultural sensitivity on a scale from 0 to 3: A score of 3 (High) meant the response included specific cultural details like dietary habits, known health risks for that group, or barriers to healthcare access. A score of 2 (Moderate) meant the response mentioned health disparities but did not provide details. A score of 1 (Low) meant very little cultural consideration. A score of 0 (None) meant the response gave generic medical advice with no cultural context. To validate this AI-based scoring methodology, each LLM response underwent independent clinical review by a team of ophthalmologists, consisting of retina and corneal specialists. The ophthalmologists evaluated the responses for cultural appropriateness, medical accuracy, and demographic sensitivity.

We used *t*-tests to find significant differences between the AI models, considering results with *p*-values less than 0.05 as statistically significant. We recorded each LLMs’ response time. Longer response times may cause users to lose interest and not read the complete answer from the LLM.

## 3. Results

**Figure 1:**
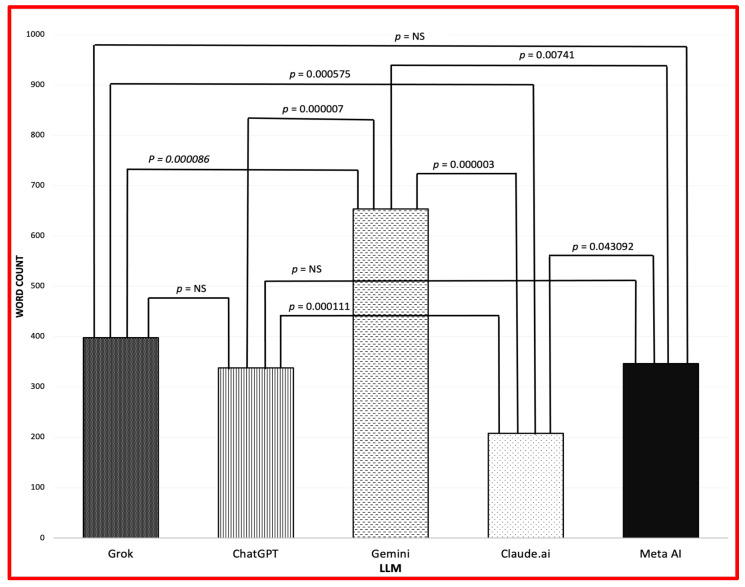
Mean word count (NS: not significant).

Figure 1 shows that Claude.ai had the briefest responses across all demographics. Gender differences in word count varied by LLM and demographic group. Grok generated 542 words for Hispanic males compared to 294 words for Hispanic females, and 266 words for Black males versus 442 words for Black females. Gemini produced 651 words for Hispanic males versus 563 words for Hispanic females, and 743 words for White males versus 591 words for White females. Meta AI generated 133 words for Black males compared to 261 words for Black females. The remaining LLMs fell within 70 words when comparing male to female responses.

**Figure 2:**
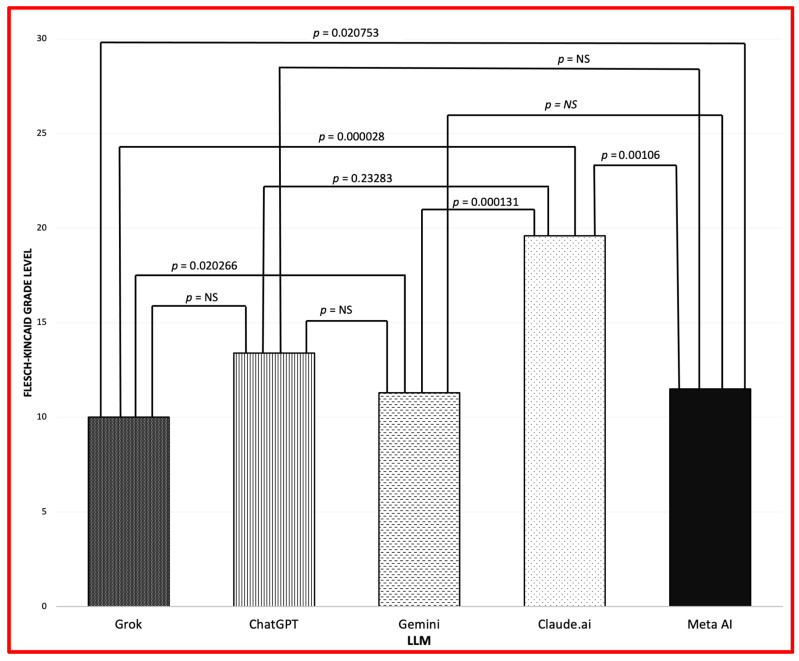
Mean Flesch-Kincaid grade level (NS: not significant).

Figure 2 shows that Claude.ai answers are at the highest grade level versus the four other LLMs, but it has the briefest responses. Thus, the user will see the facts as the same but the actual communication may entail a higher level of reading comprehension. Despite Claude.ai having the highest grade level requirement, it is ChatGPT that shows the greatest variation in the grade level in its responses.

In Table 1, there is a greater variation in word counts depending on the LLM. Claude ai has the smallest difference in word counts and it is the one with the briefest answers, but does not have the shortest time as one might expect. Gemini has the largest differential in word count and it is the wordiest of all the five LLMs studied. While Claude.ai has the highest reading level, it is ChatGPT with the greatest differential in reading level.

The CSS is a mathematical tool that measures how similar two sets of information are, regardless of their size (Table 2). Consider that there are two vectors, which can be thought of as arrows pointing in different directions. Cosine similarity calculates the cosine of the angle between these arrows. If they are pointing in a similar direction, that means that their data or sentences are similar. Each piece of information is an arrow or vector. For example, if one is comparing two medical documents, the words and concepts can be considered arrows or vectors.

If they point in completely different directions, they are not similar at all.

The CSS ranges from −1 to 1. A score of 1 means the arrows are perfectly aligned and that the information is identical or extremely similar. A score of 0 means the arrows are at right angles, representing no similarity. A score of −1 indicates that the information is completely opposite. If they point in the exact same direction, their beams overlap perfectly (cosine similarity = 1).

In medicine, CSS can help recommend medications based on a patient’s symptoms and medical history by finding the drugs with descriptions or effects most similar to the patient’s profile. Cosine similarity is a tool used to measure how similar items are, based on the alignment of their characteristics. AI models often use it to compare the “meaning” of text, including medical reports, research papers, or patient notes. This concept helps to understand how technology makes connections and provides insights in medicine.

In Table 3, there were no mentions of thyroid playing a role in dry eye except for Black females and White females in Gemini. Excessive eye medication was not mentioned by any LLMs and biotissue as a treatment option was not mentioned by any of the LLMs. All four LLMs included referrals to ophthalmologists. Dry eye syndrome was the following most mentioned keyword, except by Meta AI (Table 3). Allergies as a cause of dry eye syndrome were seen in all LLM responses except Gemini for White males, White females, Black males, and East Asian females (Table 3).

Of note, in Table 4, the shortest word count was Claude but the shortest response time was Meta. This is interesting because Gemini has the longest average word count and its response time was the longest.

Figure 1 and Figure 2 shows word count and Flesch-Kincaid Grade Level. ChatGPT-4.5 determined cultural sensitivity via AI scoring. Variations in response length were observed across LLMs (Figure 1). *t*-test results revealed significant differences between most LLM pairs for word count and reading level metrics (Figure 1 and Figure 2).

The delta word count included 32 to 346 words for all LLMs (Table 1). Meta AI showed the highest variability with a delta of 346 words, suggesting inconsistent response patterns across user demographics (Table 1).

The delta in Flesch-Kincaid scores, specifically Gemini (2.0) and ChatGPT (16.6), reveals marked inconsistencies in text complexity across LLMs, potentially affecting patient comprehension of AI-generated medical information (Table 1).

Cultural sensitivity scores across the five LLMs revealed variation in their ability to provide culturally tailored healthcare information. Gemini achieved the highest mean cultural sensitivity score (2.5 ± 0.8), followed closely by Grok (2.3 ± 0.9), while ChatGPT demonstrated moderate cultural awareness (1.8 ± 0.9). Claude.ai showed lower cultural sensitivity (1.3 ± 0.5), and Meta AI exhibited the poorest cultural tailoring with a mean score of only 0.5 ± 0.5, indicating predominantly generic responses with minimal demographic-specific considerations.

CSS indicated moderate to high content similarity across LLMs, with a total mean similarity of 75.3 ± 3.3 (Table 2).

All models recommended eye care specialist consultation across all demographic groups (Table 3). Dry eye syndrome was mentioned across all racial groups except for Meta AI. “Biotissue” received minimal mention across all models. Ocular rosacea, meibomian gland dysfunction, and thyroid disease were not mentioned often (Table 3).

Response time analysis demonstrated that Meta AI had the fastest response times with a mean of 7.41 s, followed by Claude.ai at 10.54 s (Table 4). Gemini was the slowest, with a mean time of 25.32 s (Table 4).

## 4. Discussion

### 4.1. Cultural Sensitivity Disparities Across LLMs

#### 4.1.1. Keywords

All four LLMs included referrals to ophthalmologists, with the only uniform keyword being “see an Eye MD/DO or ophthalmologist.” Meta AI excluded dry eye syndrome for Black females, likely reflecting training data limitations rather than racial predilection, since dry eye syndrome affects all populations equally.

Critical omissions were concerning. MGD, affecting up to 86% of dry eye patients, was mentioned inconsistently across models with complete omissions for multiple demographic groups [40]. Sjögren’s syndrome, affecting 0.5–1% of the population, was absent from responses to Black and Hispanic males across several LLMs [41]. These omissions have direct clinical consequences, potentially leading to treatment delays and missed opportunities for early detection of systemic diseases that require coordinated care.

#### 4.1.2. Gender and Age Considerations

Managing dry eye in 62-year-old patients requires an understanding of age-related changes, however, LLMs showed significant disparities in addressing these considerations. Most LLMs failed to mention post-menopausal hormonal changes, despite estrogen deficiency contributing to a 2–3 fold increased dry eye prevalence in post-menopausal women [34]. LLMs rarely mentioned medication-induced dry eye from antihistamines, antidepressants, and beta-blockers commonly used in this population [7].

#### 4.1.3. Cultural Sensitivity Disparities

We used ChatGPT-4.5 to grade cultural sensitivity on a 0–3 scale. Grok demonstrated the highest cultural awareness among minority populations, achieving scores of 3 for Black and Latino demographics while providing, culturally specific resource recommendations, such as EyeCare America. Claude.ai and Meta AI showed limited cultural sensitivity with predominantly lower scores.

This matters clinically because African American and Hispanic populations have higher autoimmune disease prevalences, including sarcoidosis, which is also associated with dry eye syndrome [42]. East Asian populations have documented genetic predispositions to meibomian gland dysfunction requiring population-specific approaches [43]. These disparities suggest current LLM training inadequately prepares systems to provide equitable healthcare information.

### 4.2. Healthcare Access and LLM Impact

LLMs provide instant responses to medical queries regardless of geographic location or socioeconomic status, handling over 70,000 health-related questions per minute worldwide. This addresses critical healthcare shortages, particularly where the World Health Organization reports a shortage of 4.3 million healthcare workers globally.

These tools work 24/7 without appointments, fees, or insurance requirements. A study found 68% of people in underserved areas use AI chatbots before deciding to see a doctor [44]. Mobile access democratizes healthcare information, with 78% of health-related LLM questions coming from smartphones [45].

### 4.3. User Demographics and Access Patterns

Analysis of 2.3 million health-related questions revealed that 43% came from users with limited healthcare access, 31% from non-English speakers, and 26% from individuals without health insurance [46]. Grok has a significant number of “bot” users disseminating misinformation and conspiracy theories [45].

Healthcare providers must help patients navigate this space. Stanford University and Mayo Clinic have partnered with OpenAI to create internal LLMs [47], indicating the future involves partnerships between LLM companies and hospitals to create systems with verifiable medical information following clinical guidelines.

### 4.4. Training Methodologies and Their Healthcare Implications

Different training approaches create both opportunities and challenges. Open-source models may encompass diverse healthcare perspectives, but also unverified medical claims [48]. Accuracy drops by up to 40% for non-English medical questions. Each model exhibited distinct characteristics: Grok incorporated colloquial language from X-platform training, risking healthcare misinformation [45,48]. ChatGPT provided structured explanations in a conversational tone, albeit with variable complexity. Gemini provided comprehensive responses with associated websites. Claude.ai maintained brevity with safety disclaimers. Meta AI used open-source software, images and data from its Facebook (version 523.0.0), WhatsApp (version 25.20.84), Instagram (version 390.0.0.43.81) accounts.

### 4.5. Constitutional AI and Healthcare Safety

Claude.ai has a built-in constitution that maintains proper healthcare boundaries by separating educational information from medical advice. While other LLMs apply safety filters after content generation, Claude.ai integrates its constitution directly into training, embedding ethics and accuracy from the start.

These challenges highlight the complex landscape healthcare providers must navigate when considering LLM integration. Ongoing assessment of data handling practices, safety protocols, and regulatory frameworks will be essential to maintain patient trust.

### 4.6. The Four vs. Of Medical Data Management

LLMs in healthcare must manage four critical dimensions: velocity, volume, variety, and veracity [49]. They process queries at unprecedented velocity across time zones and clinical scenarios, while handling enormous volumes from vast datasets that encompass medical literature and patient interactions. LLMs demonstrate remarkable variety in processing diverse data types, including natural language symptoms and multilingual queries [49]. Most critically, veracity requires maintaining accuracy in healthcare delivery, as medical misinformation can have serious consequences.

### 4.7. Implications for Future Healthcare Delivery

LLM improvement requires healthcare provider input during development and fine-tuning phases. Training LLMs using approved clinical guidelines from medical associations would reduce information gaps. All LLMs currently offer free trials to physicians and students, with API opportunities for physician input.

As LLMs become integrated into healthcare information seeking, ensuring equitable, accurate, and culturally sensitive responses becomes essential for improving health outcomes. In this study, LLMs generated standard treatment plans but occasionally made errors of omission. Future use should involve consulting multiple LLMs for queries, as LLM improvements from 2023 to 2024 have led to better answers [50].

### 4.8. Study Limitations

Several limitations should be considered. The single-query methodology may not capture full LLM response variability and its ability to hallucinate with repeated queries. Manual response time measurements introduce potential error. Using ChatGPT-4.5 to evaluate cultural sensitivity introduces potential AI bias versus human bias [32].

The study’s focus on a single medical condition and age group limits broader applicability. Sample size constraints prevent assessment of multiple interactions. Future research should incorporate human expert evaluations, physician-led teams with their APIs inserted into an LLM, larger sample sizes, and clinical accuracy assessment against established medical guidelines.

## 5. Conclusions

Large language models represent both a significant opportunity and challenge in dry eye patient education. As patients increasingly turn to AI-powered platforms for healthcare information, understanding the capabilities and limitations of these systems becomes critical for maintaining quality patient care.

This study demonstrates that while all LLMs consistently recommended consultation with eye care professionals, disparities exist in cultural sensitivity, content depth, and clinical comprehensiveness. The variability in how different models approach dry eye management reveals important gaps that have direct implications for patient outcomes. Most concerning is the inconsistent acknowledgment of key etiological factors, such as autoimmune diseases, hormonal changes, thyroid dysfunction, meibomian gland dysfunction, and Sjögren’s, despite these being well-established components of comprehensive dry eye evaluation [7].

These errors of omission pose significant clinical risks. For patients in underserved areas or those with limited access to specialized care, incomplete information from LLMs may result in delayed diagnosis, inadequate treatment approaches focused solely on symptomatic relief, and missed opportunities for early detection of underlying systemic conditions. For example, African Americans were not told by all LLMs about uveitis or collagen vascular disease as a cause for dry eye syndrome, yet many African Americans have sarcoidosis. Thus, specific populations may receive inadequate guidance despite facing distinct disease patterns and unique barriers to healthcare access.

The cause of these limitations lies in training methodologies that rely on internet-derived data, which can perpetuate or amplify existing medical biases. This is particularly problematic in culturally complex situations where a nuanced understanding of population-specific risk factors is essential for appropriate care recommendations. While LLMs demonstrate value in providing accessible basic health information and encouraging appropriate specialist consultation, our findings suggest that they cannot replace the clinical judgment of trained healthcare professionals.

Moving forward, the integration of LLMs into healthcare information delivery requires active physician oversight and strict alignment with evidence-based clinical guidelines from professional organizations such as the American Academy of Ophthalmology. The future of AI-assisted patient education relies on collaborative efforts among healthcare systems, physicians, technology developers, and policymakers to address these systematic biases and ensure equitable, accurate delivery of healthcare information across all demographic groups.

As dry eye syndrome continues to affect millions of patients worldwide, ensuring that AI-powered educational tools provide comprehensive, culturally sensitive, and clinically accurate information becomes essential for supporting informed patient decision-making and optimal health outcomes.

## Figures and Tables

**Table 1:** Range word count and Flesch-Kincaid readability.

LLM	ΔWC	ΔFK
Grok	276	3.1
ChatGPT	175	16.6
Gemini	288	2.0
Claude.ai	32	7.8
Meta AI	346	4.0

**Table 2:** Cosine similarity scores for each LLM.

Cosine Similarity Scores										
Race	Grok vs. ChatGPT	Grok vs. Gemini	Grok vs. Claude.ai	Grok vs. Meta AI	ChatGPT vs. Gemini	ChatGPT vs. Claude.ai	ChatGPT vs. Meta AI	Gemini vs. Claude.ai	Gemini vs. Meta AI	Claude.ai vs. Meta AI
WM	76	78	77.1	72.7	75.3	75.1	72.8	75	75.8	74.1
WF	77.2	80.3	77.1	77.3	76.6	75.1	73.9	76.9	77.7	73.9
AM	76.5	78.4	77.2	66.9	78.1	74.9	64.4	73.8	63.7	65.9
AF	78.2	78.9	73.3	70.7	79.3	74.4	69.2	78.5	70.4	75.2
EAM	75.7	78.8	77.5	77.4	76.7	72.1	71.8	77	77.6	74.3
EAF	79.4	78	76.2	77.4	78.6	74.3	74.1	75.7	75.4	72.4
HM	77.1	78.9	74.8	75.9	79.6	73.6	76.7	75.5	79.5	75.6
HF	73.7	77.3	78.3	75.6	76.4	72.7	72	76.4	73.2	73.0
Mean ± SD	76.7 ± 1.7	78.6 ± 0.9	76.4 ± 1.6	74.2 ± 3.8	77.6 ± 1.5	74.0 ± 1.1	71.9 ± 3.7	76.1 ± 1.4	74.2 ± 5.1	73.1 ± 3.1
Total Mean ± SD	75.3 ± 3.3									

WM = White Male; WF = White Female; BM = Black Male; BF = Black Female; EAM = East Asian Male, EAF = East Asian Female; HM = Hispanic Male; HF = Hispanic Female.

**Table 3:** Keyword analysis patterns.

Keywords	LLMs				
	Grok	ChatGPT	Gemini	Claude.AI	Meta AI
Eye MD/DO or Ophthalmologist	**+**	**+**	**+**	**+**	**+**
Dry Eye Syndrome	**+**	**+**	**+**	**+**	**+** Except BF
Sjögren/Uveitis/Collagen vascularity disease	**+** Except BM, BF, HM	**+** Except HF	**+**	**+** Except BM, BF, HF	-
Allergy	**+**	**+**	**+** Except WM, WF, EAF, BM	**+**	**+**
Meibomian Gland Dysfunction	- Except WM, EAF	- Except WM, EAM	- Except EAM	- Except WM, WF, HF	-
Thyroid	-	-	- Except WF, BF	-	-
Excessive Eye Meds	-	-	-	-	-
Biotissue	-	-	-	-	-
Ocular Rosacea	-	**+** Except EAM	-	-	-

(-) = No LLMs Mentioned; (+) = All LLMs Mentioned.

**Table 4:** LLMs’ response generation times.

Race	LLM Response Time (Seconds)				
	Grok	ChatGPT	Gemini	Claude.AI	Meta AI
WM	13.51	22.72	24.44	11.02	7.71
WF	12.97	23.78	20.8	8.37	7.97
BM	11.97	18.68	27.27	10.78	3.3
BF	12.21	19.72	18.77	13.53	4.76
EAM	11.13	16.03	32.87	10.69	7.47
EAF	21.82	15.52	23.47	10.26	9.12
HM	16.17	17.82	28.62	9.73	8.91
HF	11.01	15.45	26.35	9.91	10.07
Mean	13.85	18.72	25.32	10.54	7.41
Range	10.81	8.33	14.1	5.16	6.77

WM = White Male; WF = White Female; BM = Black Male; BF = Black Female; EAM = East Asian Male, EAF = East Asian Female; HM = Hispanic Male; HF = Hispanic Female.

## Data Availability

Dataset available on request from the authors: The raw data supporting the conclusions of this article will be made available by the authors on request.

## Abbreviations

The following abbreviations are used in this manuscript:

MGD	Meibomian Gland Dysfunction
LLM	Large Language Model
Llama	Large Language Model Meta AI
RLHF	Reinforcement Learning from Human Feedback
AI	Artificial Intelligence
PPO	Proximal Policy Organization
PaLM 2	Pathways Language Model 2
FK	Flesch-Kincaid Grade Level
CSS	Cosine Similarity Score
